# Co-Occurrence of Lifestyle Risk Behaviors Among Physical Education and Sport University Students: Evidence from a Cluster Analysis

**DOI:** 10.3390/healthcare14091145

**Published:** 2026-04-24

**Authors:** Vanessa Santos, Joana Serpa, Mariana Parreira, Vanda Correia, Priscila Marconcin

**Affiliations:** 1 Exercise and Health Laboratory, Interdisciplinary Center for the Study of Human Performance (CIPER), Faculty of Human Kinetics, University of Lisbon, 1649-004 Lisboa, Portugal; 2 Insight: Piaget Research Center for Ecological Human Development, Instituto Piaget, 2805-059 Almada, Portugal; joana.serpa@ipiaget.pt (J.S.); priscila.marconcin@ipiaget.pt (P.M.); 3 Higher Institute of Intercultural and Transdisciplinary Studies (ISEIT) Almada, Instituto Piaget, 2805-059 Almada, Portugal; 2023115363@ipiaget.pt; 4 Sport Physical Activity and Health Research & Innovation Center (SPRINT), 2040-413 Rio Maior, Portugal; vicorreia@ualg.pt; 5 Higher School of Education and Communication, Universidade do Algarve, 8005-139 Faro, Portugal; 6 Spots Expertise Laboratory, Interdisciplinary Center for the Study of Human Performance (CIPER), Faculty of Human Kinetics, University of Lisbon, 1649-004 Lisboa, Portugal; 7 Faculty of Health Sciences, Universidad Autónoma de Chile, Providencia 7500912, Chile

**Keywords:** university students, lifestyle behaviors, alcohol consumption, smoking, sedentary behavior, cluster analysis

## Abstract

**Highlights:**

**What are the main findings?**

- Lifestyle risk behaviors clustered into three distinct profiles among Physical Education and Sport university students.
- Alcohol consumption was highly prevalent across clusters, while smoking was concentrated in a smaller high-risk subgroup.

**What are the implications of the main findings?**

- Health promotion strategies in university settings should target multiple co-occurring behaviors rather than isolated risk factors.
- Physically active students may still present significant sedentary behavior and other risk factors, highlighting the need for comprehensive lifestyle interventions.

**Abstract:**

**Background**: Health-related behaviors often cluster during young adulthood, potentially increasing the risk of long-term adverse health outcomes. Understanding how lifestyle risk behaviors co-occur among university students is essential for developing targeted health promotion strategies. **Objective**: This study aimed to identify lifestyle risk profiles among university students based on the co-occurrence of smoking behavior, alcohol consumption, sedentary behavior, and body weight status. **Methods**: A cross-sectional study was conducted with 147 university students enrolled in a physical education and sport undergraduate program (mean age: 20.58 ± 2.94 years; 80.3% male). Physical activity and sedentary behavior were assessed using the International Physical Activity Questionnaire–Short Form (IPAQ-SF), while smoking and alcohol consumption were self-reported. Body mass index was used to classify weight status. Lifestyle risk profiles were identified using two-step cluster analysis based on regular smoking, alcohol consumption, sedentary behavior, and overweight/obesity. Differences in cluster distribution according to sex and federated athlete status were examined using chi-square tests. A two-step cluster analysis based on the Bayesian Information Criterion (BIC) and silhouette measure was used to identify lifestyle risk profiles. **Results**: Overall, 46.9% of participants had experimented with tobacco, 11.6% were current smokers, and 74.8% reported alcohol consumption. Participants accumulated an average of 3772.25 ± 1957.99 MET-min/week of physical activity. Three distinct lifestyle risk profiles were identified. Cluster 1 (46.9%), labeled the alcohol profile, was characterized by alcohol consumption without smoking and no prevalence of being overweight. Cluster 2 (20.4%), the multiple-risk profile, included participants who reported regular smoking, with nearly half presenting sedentary behavior and overweight/obesity. Cluster 3 (32.7%), the overweight profile, was characterized by overweight/obesity combined with alcohol consumption but no smoking. No significant differences were observed in the distribution of lifestyle profiles according to sex (*p* = 0.111) or federated athlete status (*p* = 0.087). **Conclusions**: Lifestyle risk behaviors cluster into distinct profiles among university students, with alcohol consumption appearing across multiple profiles and smoking concentrated in a specific high-risk group. These findings highlight the need for targeted health promotion strategies addressing multiple co-occurring behaviors within university populations.

## 1. Introduction

The adoption of healthy lifestyle behaviors during young adulthood is a key determinant of long-term health outcomes [1]. This life stage represents a critical window for the establishment of health-related habits that may persist throughout adulthood and significantly influence the risk of non-communicable diseases [2,3]. University years represent a transitional period characterized by increased independence, changes in daily routines, and new academic and social demands, which may influence health-related behaviors [4]. During this stage, students may develop or consolidate habits related to alcohol consumption, smoking, physical activity, sedentary behavior, and dietary patterns, many of which may persist into later adulthood and contribute to the development of chronic diseases [5]. Several studies have shown that lifestyle behaviors established during early adulthood tend to track into later life and significantly influence long-term cardiometabolic health outcomes [6,7,8].

Among these behaviors, regular physical activity plays a central role in promoting physical and mental health, contributing to the prevention of cardiovascular diseases, obesity, metabolic disorders, and psychological distress [9,10]. Conversely, insufficient physical activity and prolonged sedentary behaviour have been associated with increased cardiometabolic risk and poorer health outcomes among young adults [11,12,13]. In particular, recent research highlights the coexistence of high levels of physical activity with prolonged sedentary time, a pattern increasingly observed among university students. These behaviors are considered independent, meaning that high levels of physical activity do not necessarily offset the negative effects of prolonged sedentary behavior [11,13,14]. Despite the well-established benefits of regular physical exercise, several studies have shown that university students frequently engage in unhealthy behaviors, including excessive alcohol consumption, tobacco use, and prolonged sitting time [14,15,16].

In addition to physical activity and sedentary behavior, other health risk behaviors such as tobacco use, alcohol consumption, and excess body weight are highly prevalent among young adults and contribute significantly to the development of non-communicable diseases [5,14,15,16]. These behaviors are often influenced by social and environmental factors, particularly within university settings, where lifestyle habits may be shaped by peer interactions, academic demands, and changes in daily routines [17,18,19]. Health risk behaviors rarely occur in isolation. Instead, these behaviors often co-occur and cluster within individuals, forming distinct behavioral profiles that may amplify the risk of negative health outcomes [17,18]. Previous research has demonstrated that behaviors such as alcohol consumption, smoking, physical inactivity, and sedentary lifestyles frequently appear together, particularly among young adults [17,18,19]. Understanding how these behaviors cluster is essential because individuals exposed to multiple concurrent risk factors are at a substantially higher risk of developing chronic diseases later in life [17].

University students represent an important population for examining the clustering of lifestyle risk behaviors [14,15]. However, evidence regarding how multiple lifestyle behaviors cluster specifically among students enrolled in physical education and sport programs remains limited. Although students enrolled in physical education and sport-related programs are often expected to present healthier lifestyles due to their academic training and involvement in sports, evidence suggests that engagement in sports or regular physical activity does not necessarily prevent the adoption of other unhealthy behaviors, such as alcohol consumption or smoking [15,20]. Consequently, examining lifestyle patterns in this population may provide important insights into the complexity of health-related behaviors among physically active young adults.

Traditional research has frequently examined health behaviors independently; however, this approach may overlook the complex interactions between multiple behaviors. Analytical techniques such as cluster analysis allow the identification of naturally occurring lifestyle profiles within populations, offering a more comprehensive understanding of how risk behaviors co-exist [14]. This analytical approach enables the identification of groups of individuals who share similar behavioral patterns, providing valuable insights into how multiple lifestyle risk factors interact within real-world populations. Identifying such profiles may help guide interventions aimed at addressing multiple health risk behaviors simultaneously and contribute to the development of more targeted health promotion strategies in university settings.

Although previous studies have explored lifestyle behaviors among university students, limited evidence exists regarding whether these patterns differ according to sex and engagement in organized sports. Considering that biological, social, and behavioral differences may influence health-related behaviors, and that participation in federated sports may act as either a protective or risk factor, it is important to explore how these variables relate to lifestyle risk profiles.

Therefore, the present study aimed to identify lifestyle risk profiles among university students based on the co-occurrence of smoking behavior, alcohol consumption, sedentary behavior, and body weight status. Additionally, the study explored whether the distribution of these lifestyle profiles differed according to sex and federated athlete status among students enrolled in a Physical Education and Sport undergraduate program.

The present study was guided by two research questions: (i) whether lifestyle risk behaviors co-occur in distinct patterns among Physical Education and Sport university students, and (ii) whether the distribution of these profiles differs according to sex and federated athlete status. Based on the previous literature, we expected to identify distinct lifestyle risk profiles and explored whether their distribution varied by sex and federated athlete status.

## 2. Materials and Methods

### 2.1. Study Design and Participants

This cross-sectional study was conducted with a convenience sample of undergraduate students enrolled in the Physical Education and Sport program at a university in *Almada*, *Portugal*. Given the exploratory nature of the study and the use of cluster analysis to identify behavioral patterns within a specific university population, a convenience sample was considered appropriate for this purpose. However, the sample size and non-probabilistic recruitment strategy may limit the representativeness and generalizability of the findings. Participants were recruited among students enrolled in the first, second, and third years of the undergraduate program, according to their current academic registration. All students present during the scheduled classes at the time of data collection were invited to participate. Participation was voluntary. All completed questionnaires were included in the final analysis. The inclusion criteria were (i) being enrolled in the undergraduate program in Physical Education and Sport and (ii) actively attending classes during the data collection period. Exclusion criteria included (i) refusal to participate in the study and (ii) submission of incomplete or inconsistent questionnaires that prevented the accurate assessment of the variables under study. Data were collected in December 2025 through self-administered questionnaires assessing sociodemographic characteristics and health-related behaviors. The questionnaires were administered in classroom settings in groups of up to 25 students per session. The questionnaire was administered in paper-based format. Prior to data collection, participants received a brief explanation of the study objectives and procedures, and informed consent was obtained before completing the questionnaire. Participants completed the questionnaires individually, while three researchers were present to provide clarification and ensure standardized administration procedures. Participation was voluntary, and responses were collected anonymously to ensure confidentiality.

This study is part of a broader research project investigating lifestyle behaviors and health-related risk factors among university students and received approval from the Institutional Ethics Committee (CEUAlg Pnº135/2025) in accordance with the ethical standards of the Declaration of Helsinki.

### 2.2. Measures

#### 2.2.1. Sociodemographic Variables

Participants reported their age, sex, academic year, and federated athlete status.

#### 2.2.2. Body Mass Index (BMI)

Body mass index (BMI) was calculated from self-reported weight and height and classified according to standard categories. For the cluster analysis, BMI was dichotomized into 0 = normal weight and 1 = overweight/obesity.

#### 2.2.3. Smoking Behavior

Smoking behavior was assessed through self-reported questions regarding tobacco experimentation and current smoking status. Participants were asked whether they had ever tried smoking and whether they currently smoked. For the cluster analysis, smoking variables were coded as binary variables: smoking experimentation (0 = never; 1 = at least once); regular smoking (0 = no; 1 = yes).

For the cluster analysis, only the variable “regular smoking” (0 = no; 1 = yes) was included, while smoking experimentation was used for descriptive purposes only.

#### 2.2.4. Alcohol Consumption

Participants reported their alcohol consumption frequency and the typical number of alcoholic drinks consumed per occasion. For analytical purposes, alcohol consumption was categorized as 0 = does not consume alcohol and 1 = consumes alcohol. Alcohol consumption was dichotomized for the purposes of cluster analysis to ensure consistency with other binary risk indicators.

#### 2.2.5. Physical Activity and Sedentary Behavior

Physical activity and sedentary behavior were assessed using the International Physical Activity Questionnaire–Short Form (IPAQ-SF) [21]. The IPAQ-SF consists of 7 items assessing the frequency and duration of walking and moderate and vigorous physical activity, as well as sitting time over the previous 7 days. Total weekly physical activity was calculated in MET-minutes per week, following the IPAQ scoring protocol. Participants were classified as either active (≥600 MET-min/week) or inactive (<600 MET-min/week) according to WHO physical activity recommendations [10]. Sedentary behavior was estimated based on the reported time spent sitting on weekdays and weekends. Average daily sitting time was calculated from weekday and weekend sitting time, and sedentary behavior was categorized as 0 = non-sedentary (<8 h/day) and 1 = sedentary (≥8 h/day), consistent with previous sedentary behaviour research [11].

Physical activity was not included as a clustering variable due to the lack of variability in the sample, as the vast majority of participants were classified as physically active. Therefore, sedentary behavior was considered a more informative indicator of lifestyle-related risk in this population.

#### 2.2.6. Risk Behavior Indicators

Four binary lifestyle risk indicators were included in the analysis: overweight/obesity, regular smoking, alcohol consumption and sedentary behavior. The number of risk behaviors was calculated for each participant, ranging from 0 to 4.

### 2.3. Statistical Analysis

Descriptive statistics were used to summarize sample characteristics and health-related behaviors. Continuous variables are presented as mean and standard deviation, while categorical variables are presented as frequencies and percentages. A two-step cluster analysis was conducted to identify lifestyle risk profiles among university students based on the four binary risk indicators: overweight/obesity, regular smoking, alcohol consumption, and sedentary behavior. This method allows the identification of natural groupings within the data and is suitable for datasets containing categorical variables. Model selection was based on the Bayesian Information Criterion (BIC) and the silhouette measure of cohesion and separation, together with the interpretability of the resulting clusters. The final solution identified three distinct clusters representing different lifestyle risk profiles. Differences in cluster distribution according to sex and federated athlete status were examined using chi-square tests. Statistical significance was set at *p* < 0.05. The analyses were performed using IBM SPSS Statistics version 19 (IBM Corp., Armonk, NY, USA).

## 3. Results

A total of 147 university students participated in the study. Most participants were male (80.3%), and the mean age was 20.58 ± 2.94 years. Regarding academic year, 38.1% were first-year students, 27.9% were in the second year, and 34.0% were in the third year. Additionally, 37.4% of the participants were federated athletes, and the majority (87.1%) reported regular sports practice. The mean body mass index (BMI) of the sample was 24.59 ± 5.13 kg/m^2^ (Table 1). Regular sports practice was defined as engagement in structured or unstructured sports or exercise activities at least two times per week for a minimum of 30 min per session.

The distribution of health risk behaviors, physical activity, and sedentary behavior is presented in Table 2. Nearly half of the participants (46.9%) reported having tried smoking at least once, although the majority (88.4%) were not current smokers. Daily smoking was reported by 4.8%, and 6.8% reported smoking occasionally. Regarding electronic cigarette use, most students (89.1%) reported no use, while 3.4% reported daily use and 7.5% occasional use. Overall, 11.6% of participants were classified as current smokers (daily or occasional), ensuring consistency with the reporting of alcohol consumption.

Alcohol consumption was common among participants, with 40.1% reporting drinking alcohol two or more times per month, and 50.3% reporting consuming one to two drinks per occasion. Total physical activity was further categorized into low (<600 MET-min/week), moderate (600–3000 MET-min/week), and high (>3000 MET-min/week), according to IPAQ guidelines. Participants reported an average sitting time of 515.34 ± 172.90 min/day on weekdays and 512.84 ± 178.79 min/day on weekend days. The mean total physical activity level was 3772.25 ± 1957.99 MET-min/week.

The distribution of the coded lifestyle risk behaviors used in the cluster analysis is presented in Table 3. Overall, 42.2% of participants were classified as overweight or obese, while 11.6% reported regular smoking. Alcohol consumption was reported by 74.8% of the sample. Regarding physical activity levels, the vast majority of participants (98.0%) were classified as physically active, with only 2.0% categorized as inactive. In terms of sedentary behavior, 48.3% of participants were classified as sedentary, while 51.7% were considered non-sedentary.

### 3.1. Co-Occurrence of Lifestyle Risk Behaviors

The distribution of the number of lifestyle risk behaviors among participants showed that 4.1% reported no risk behaviors, 25.2% reported one risk behavior, 46.9% reported two risk behaviors, 19.7% reported three risk behaviors, and 4.1% reported four co-occurring risk behaviors. On average, male students presented 1.98 ± 0.89 risk behaviors, whereas female students presented 1.79 ± 0.81 risk behaviors, with no statistically significant differences between sexes (*p* = 0.300). Similarly, the mean number of risk behaviors did not differ significantly according to federated athlete status, with federated athletes presenting 1.89 ± 0.80 risk behaviors and non-athletes 1.97 ± 0.92 risk behaviors (*p* = 0.563).

The lifestyle risk behavior profiles identified through cluster analysis are presented in Table 4. Although sedentary behavior was prevalent across all clusters, it did not define a distinct cluster profile, as its distribution was relatively similar among groups, preventing it from emerging as a primary differentiating variable in the clustering solution. The analysis revealed three distinct clusters among university students. Cluster 1 (Alcohol profile; n = 69) was characterized by alcohol consumption in all participants (100%), with no regular smoking and no cases of overweight or obesity, although 47.8% were classified as sedentary. Cluster 2 (Multiple-risk profile; n = 30) represented the highest-risk group, as it combined multiple risk behaviors, including 100% regular smoking, 46.7% overweight/obesity, 46.7% sedentary behavior, and 53.3% alcohol consumption. Cluster 3 (Overweight profile; n = 48) was characterized by overweight or obesity in all participants (100%), with alcohol consumption also reported by all individuals (100%), no regular smoking, and 50.0% classified as sedentary.

### 3.2. Distribution of Risk Profiles

The distribution of lifestyle risk profiles according to sex and federated athlete status is presented in Table 5. No significant differences were observed in the distribution of lifestyle risk profiles according to sex (χ^2^ = 4.39, *p* = 0.111) or federated athlete status (χ^2^ = 4.88, *p* = 0.087). However, a lower proportion of federated athletes was observed in the multiple-risk cluster, suggesting a tendency for this group to be less represented among athletes. Overall, these findings indicate that lifestyle risk behaviors were relatively homogeneously distributed across the sample, regardless of sex or athlete status.

## 4. Discussion

The findings of this study indicate that lifestyle risk behaviors among university students tend to organize into distinct behavioral patterns rather than occurring independently. The cluster analysis identified three main lifestyle risk profiles: an alcohol profile, a multiple-risk profile, and an overweight profile. These patterns suggest that health-related behaviors in young adults are interconnected and may reflect shared social, environmental, and lifestyle influences characteristic of the university context [17,22]. Previous research has consistently shown that health behaviors such as alcohol consumption, smoking, physical inactivity, and unhealthy diet often cluster together during young adulthood [17,18,20]. This clustering phenomenon is consistent with the concept of multiple risk behavior accumulation, which suggests that health-related behaviors are shaped by common determinants such as social norms, environmental contexts, and lifestyle routines during the transition to adulthood. These shared determinants may contribute to the development of behavioral patterns that reinforce each other over time, increasing the likelihood of cumulative health risks [23]. Understanding these behavioral profiles is particularly important for identifying groups of students who may be more vulnerable to cumulative health risks and for guiding more comprehensive health promotion strategies within higher education settings.

Alcohol consumption emerged as the most prevalent behavior across the identified clusters, being present in both the alcohol profile and the overweight profile. This finding is consistent with previous research indicating that alcohol consumption is highly prevalent among university students and often represents a central component of student social culture. Studies conducted in different countries have reported similarly high levels of alcohol use in university populations, frequently associated with socialization contexts and academic life transitions [14,15,16]. This pattern may reflect the normalization of alcohol consumption within university environments, where social activities and peer interactions frequently involve drinking behaviors [15,16].

The cluster analysis also identified a smaller subgroup characterized by the simultaneous presence of multiple risk behaviors, including smoking, overweight/obesity, and sedentary behavior. This multiple-risk profile represents the group with the highest potential health vulnerability, as the accumulation of risk factors may increase the likelihood of developing chronic diseases later in life. Previous studies have consistently shown that individuals who engage in smoking are more likely to present additional unhealthy behaviors, such as low physical activity, excessive alcohol consumption, and poor dietary habits [17,18,19]. This co-occurrence suggests reflecting shared behavioral and psychosocial determinants, including lifestyle routines, stress management strategies, and social influences that shape health behaviors during young adulthood. These findings reinforce the importance of considering multiple health behaviors simultaneously when designing preventive strategies for young adult populations [17]. The identification of this cluster highlights the importance of addressing multiple behaviors simultaneously in health promotion programs targeting university populations.

Another relevant finding of the present study was the identification of a cluster characterized by overweight or obesity combined with alcohol consumption but without regular smoking. This pattern suggests that even among students who do not smoke, other lifestyle factors may still contribute to increased health risk. Alcohol consumption has been associated with increased caloric intake and weight gain, which may partly explain the co-occurrence observed in this cluster. Similar associations between alcohol consumption and overweight have been reported in studies examining lifestyle patterns in young adults [14,16]. Additionally, alcohol-related caloric intake combined with sedentary leisure activities may contribute to positive energy balance and subsequent weight gain in young adult populations [24].

Despite the relatively high levels of sedentary behavior observed in the sample, most participants were classified as physically active, with an average physical activity level well above the recommended threshold. This finding is not unexpected given that the sample consisted of students enrolled in a Physical Education and Sport program, who are typically more engaged in structured physical activity. Nevertheless, the presence of sedentary behavior in nearly half of the participants highlights the coexistence of high physical activity and prolonged sitting time, a phenomenon increasingly described in the literature as the “active but sedentary” lifestyle pattern. This pattern has important health implications, as sedentary behavior has been identified as an independent risk factor for cardiometabolic diseases and mortality, even among individuals who meet physical activity recommendations [13]. Therefore, reducing sedentary time should be considered a complementary target alongside promoting physical activity. This finding is consistent with current public health guidelines, which emphasize sedentary behavior as an independent health risk factor [25]. Previous research suggests that high levels of sedentary behavior may still pose health risks even among physically active individuals [11,13]. This suggests that engaging in recommended levels of physical activity may not fully compensate for the potential health risks associated with prolonged sedentary time [11].

The study also examined whether lifestyle risk profiles differed according to sex and federated athlete status. No statistically significant differences were observed in the distribution of clusters across these variables. These findings suggest that lifestyle risk behavior profiles were evenly distributed across the sample, regardless of sex or participation in federated sports. However, the interpretation of these findings should be considered with caution given the sample sex imbalance (predominantly male).

According to the WHO, women tend to be less physically active than men [25], and the high levels of physical activity observed in the present study may partly reflect the sex distribution of the sample, in which males represented 80.3% of participants. However, no statistically significant differences were observed between sexes (*p* = 0.111).

Similarly, although athletes are often expected to demonstrate healthier behaviors, the present findings showed no statistically significant differences in cluster distribution according to federated athlete status (*p* = 0.087). This result is consistent with the previous literature suggesting that participation in organized sports does not necessarily prevent engagement in other risk behaviors, particularly alcohol consumption [26,27]. Nevertheless, the lower proportion of federated athletes observed in the multiple-risk cluster may suggest a potential protective influence of sports participation, although this trend did not reach statistical significance.

The present findings have important implications for health promotion strategies within university settings. Traditional health promotion approaches often target single behaviors, such as smoking cessation or physical activity promotion. However, the clustering of lifestyle risk behaviors observed in this study suggests that interventions addressing multiple behaviors simultaneously may be more effective. Universities represent a strategic environment for implementing such programs, as they provide opportunities to promote healthy lifestyles through academic curricula, sports programs, and campus-wide health initiatives. Addressing multiple health behaviors simultaneously may therefore enhance the effectiveness of preventive strategies aimed at improving long-term health outcomes among university students. Integrating health promotion initiatives within university environments may therefore contribute to the early prevention of chronic diseases by encouraging healthier lifestyle patterns among young adults.

Several limitations should be considered when interpreting the results of this study. First, the cross-sectional design prevents the establishment of causal relationships between the observed behaviors. Second, the study relied on self-reported data, which may be subject to recall and social desirability bias, particularly for behaviors such as alcohol consumption and smoking. Although anonymity and confidentiality were ensured, the possibility of underreporting or misreporting cannot be excluded. Third, the sample consisted of students enrolled in a Physical Education and Sport program from a single university, which may limit the generalizability of the findings to other student populations.

Additionally, some limitations related to the cluster analysis itself should be acknowledged. The relatively modest sample size may influence the stability of the cluster solution, and the results may vary if applied to larger or more diverse samples. Furthermore, the cluster analysis was based on a limited number of lifestyle risk indicators, which may not fully capture the complexity of health-related behaviors in young adults. Additionally, alcohol consumption was analyzed as a binary variable, which does not capture differences in frequency or quantity of consumption. Therefore, individuals with occasional and frequent alcohol use were classified within the same category, which may limit the sensitivity and interpretability of the findings. Other relevant variables, such as dietary habits, sleep patterns, or mental health indicators, could potentially contribute to different behavioral profiles. Although the IPAQ-SF is a widely validated and commonly used instrument, it reflects physical activity over the previous seven days and may not fully represent habitual behavior. Consequently, the results may be influenced by short-term fluctuations in activity levels.

Another aspect to consider is the imbalance in sex distribution within the sample, with a predominance of male participants, which may influence the representativeness of the identified lifestyle profiles. Future studies including more balanced samples may provide a clearer understanding of potential sex-related differences in lifestyle behavior clustering.

Future research should consider longitudinal designs and more diverse student populations to better understand the evolution and determinants of lifestyle behavior clustering over time. Despite these limitations, the use of cluster analysis allowed the identification of meaningful lifestyle patterns, providing valuable insights into the co-occurrence of health-related behaviors in this population.

From a practical perspective, interventions targeting university students should adopt a multidimensional strategy addressing multiple co-occurring behaviors simultaneously. Strategies may include combining educational programs with behavior change techniques, promoting active lifestyles while reducing sedentary time, and implementing campus-based initiatives that encourage healthy habits. Integrating these strategies within academic and social environments may enhance their effectiveness and support long-term behavior change.

## 5. Conclusions

The present study identified distinct lifestyle risk behavior profiles among university students enrolled in a Physical Education and Sport program. The findings revealed that alcohol consumption was highly prevalent across the identified clusters, while a smaller subgroup presented a multiple-risk profile characterized by the coexistence of smoking, overweight/obesity, and sedentary behavior. These results highlight that lifestyle risk behaviors among young adults tend to cluster rather than occur independently, reinforcing the importance of considering multiple behaviors simultaneously when examining health-related patterns.

Despite the high levels of physical activity observed in the sample, sedentary behavior remained prevalent, suggesting the coexistence of physically active yet sedentary lifestyles among university students. This finding emphasizes that meeting recommended physical activity levels may not fully offset the potential health risks associated with prolonged sedentary time.

Overall, these results underscore the importance of implementing health promotion strategies in university settings that address multiple lifestyle behaviors simultaneously and move beyond single-behavior interventions. Understanding how risk behaviors cluster may contribute to the development of more comprehensive interventions aimed at promoting healthier lifestyles and reducing long-term health risks among young adults.

## Figures and Tables

**Table 1:** Sample characteristics (n = 147).

Variables	Frequency (%)	Mean (SD)
Age (years)	-	20.58 (2.94)
Body mass index (kg/m^2^)		24.59 (5.13)
Sex		-
Female	29 (19.7)	
Male	118 (80.3)	
Academic Year		
1st year	56 (38.1)	-
2nd year	41 (27.9)	
3rd year	50 (34.0)	
Federated athlete		-
Yes	55 (37.4)	
No	92 (62.6)	
Regular sports practice		-
Yes	128 (87.1)	
No	19 (12.9)	

Legend: SD (standard deviation).

**Table 2:** Distribution of health risk behaviors by sex and federated athlete status (n = 147).

Variables	Total n (%)/Mean (SD)	Male	Female	Federated	Non-Federated
Smoking experimentation					
Never	78 (53.1)	63 (53.4)	15 (51.7)	35 (63.6)	43 (46.7)
Tried at least once	69 (46.9)	55 (46.6)	14 (48.3)	20 (36.4)	49 (53.3)
Current smoking (tobacco)					
No	130 (88.4)	104 (88.1)	26 (89.7)	51 (92.7)	79 (85.9)
Daily	7 (4.8)	6 (5.1)	1 (3.4)	1 (1.8)	6 (6.5)
Occasionally	10 (6.8)	8 (6.8)	2 (6.9)	3 (5.5)	7 (7.6)
Electronic cigarette use					
No	131 (89.1)	106 (89.8)	25 (86.2)	49 (89.1)	82 (89.1)
Daily	5 (3.4)	3 (2.5)	2 (6.9)	2 (3.6)	3 (3.3)
Occasionally	11 (7.5)	9 (7.6)	2 (6.9)	4 (7.3)	7 (7.6)
Alcohol consumption frequency					
Never	37 (25.2)	28 (23.7)	9 (31.0)	8 (14.5)	29 (31.5)
≤1 time/month	51 (34.7)	39 (33.1)	12 (41.4)	26 (47.3)	25 (27.2)
2–4 times/month	27 (18.4)	23 (19.5)	4 (13.8)	11 (20.0)	16 (17.4)
2–3 times/week	23 (15.6)	20 (16.9)	3 (10.3)	8 (14.5)	15 (16.3)
≥4 times/week	9 (6.1)	8 (6.8)	1 (3.4)	2 (3.6)	7 (7.6)
Number of alcoholic drinks per occasion					
1–2 drinks	74 (50.3)	59 (50.0)	15 (51.7)	29 (52.7)	45 (48.9)
3–4 drinks	24 (16.3)	20 (16.9)	4 (13.8)	12 (21.8)	12 (13.0)
5–6 drinks	16 (10.9)	13 (11.0)	3 (10.3)	7 (12.7)	9 (9.8)
7–9 drinks	8 (5.4)	7 (5.9)	1 (3.4)	2 (3.6)	6 (6.5)
≥10 drinks	11 (7.5)	9 (7.6)	2 (6.9)	2 (3.6)	9 (9.8)
Does not drink	14 (9.5)	10 (8.5)	4 (13.8)	3 (5.5)	11 (12.0)
Regular exercise/sport practice					
Yes	128 (87.1)	104 (88.1)	24 (82.8)	55 (100.0)	73 (79.3)
No	19 (12.9)	14 (11.9)	5 (17.2)	0 (0.0)	19 (20.7)
Sitting time (min/day)	Mean (SD)	Mean (SD)	Mean (SD)	Mean (SD)	Mean (SD)
Weekdays	515.34 (172.90)	519.03 (175.60)	500.34 (163.54)	536.00 (192.02)	503.00 (160.22)
Weekend days	512.84 (178.79)	508.54 (175.84)	530.34 (192.59)	498.91 (169.32)	521.17 (184.63)
Total physical activity (MET-min/week)	3772.25 (1957.99)	3694.67 (1789.67)	4087.98 (2545.20)	4219.89 (1984.46)	3504.65 (1902.76)

Legend: Values are presented as n (%) for categorical variables and mean (standard deviation) for continuous variables.

**Table 3:** Coded lifestyle risk behaviors (n = 147).

Variables	Frequency (%)
Body weight status	
0—Normal weight	85 (57.8)
1—Overweight/obesity	62 (42.2)
Regular smoking	
0—No	130 (88.4)
1—Yes	17 (11.6)
Alcohol consumption	
0—No	37 (25.2)
1—Yes	110 (74.8)
Physical activity level	
0—Active	144 (98.0)
1—Inactive	3 (2.0)
Sedentary behavior	
0—Non-sedentary	76 (51.7)
1—Sedentary	71 (48.3)

**Table 4:** Lifestyle risk behavior profiles identified by cluster analysis.

Variables	Cluster 1 Alcohol Profile (n = 69)	Cluster 2 Multiple-Risk Profile (n = 30)	Cluster 3 Overweight Profile (n = 48)
Sedentary (%)	47.8	46.7	50.0
Overweight/obesity (%)	0.0	46.7	100
Regular smoking (%)	0.0	100	0.0
Alcohol consumption (%)	100	53.3	100

**Table 5:** Distribution of Lifestyle Risk Profiles According to Sample Characteristics.

Variable	Cluster 1 n (%)	Cluster 2 n (%)	Cluster 3 n (%)	*p*-Value
Sex				
Female	18 (26.1)	6 (20.0)	5 (10.4)	0.111
Male	51 (73.9)	24 (80.0)	43 (89.6)	
Federated athlete status				
Yes	29 (42.0)	6 (20.0)	20 (41.7)	0.087
No	40 (58.0)	24 (80.0)	28 (58.3)	

## Data Availability

The data presented in this study are available on request from the corresponding author. The data are not publicly available due to privacy restrictions.

## Abbreviations

The following abbreviations are used in this manuscript:

IPAQ-SF	International Physical Activity Questionnaire–Short Form
BMI	Body Mass Index
MET	Metabolic Equivalent of Task
WHO	World Health Organization
BIC	Bayesian Information Criterion
SPSS	Statistical Package for the Social Sciences
